# Hippocampus Shape Analysis and Late-Life Depression

**DOI:** 10.1371/journal.pone.0001837

**Published:** 2008-03-19

**Authors:** Zheen Zhao, Warren D. Taylor, Martin Styner, David C. Steffens, K. Ranga R. Krishnan, James R. MacFall

**Affiliations:** 1 Neuropsychiatric Imaging Research Laboratory, Duke University Medical Center, Durham, North Carolina, United States of America; 2 Department of Biomedical Engineering, Duke University, Durham, North Carolina, United States of America; 3 Department of Radiology, Duke University Medical Center, Durham, North Carolina, United States of America; 4 Department of Psychiatry, Duke University Medical Center, Durham, North Carolina, United States of America; 5 Department of Computer Science, University of North Carolina, Chapel Hill, North Carolina, United States of America; 6 The Duke-NUS Graduate Medical School Singapore, Singapore, Singapore; James Cook University, Australia

## Abstract

Major depression in the elderly is associated with brain structural changes and vascular lesions. Changes in the subcortical regions of the limbic system have also been noted. Studies examining hippocampus volumetric differences in depression have shown variable results, possibly due to any volume differences being secondary to local shape changes rather than differences in the overall volume. Shape analysis offers the potential to detect such changes. The present study applied spherical harmonic (SPHARM) shape analysis to the left and right hippocampi of 61 elderly subjects with major depression and 43 non-depressed elderly subjects. Statistical models controlling for age, sex, and total cerebral volume showed a significant reduction in depressed compared with control subjects in the left hippocampus (F_1,103_ = 5.26; *p* = 0.0240) but not right hippocampus volume (F_1,103_ = 0.41; *p* = 0.5213). Shape analysis showed significant differences in the mid-body of the left (but not the right) hippocampus between depressed and controls. When the depressed group was dichotomized into those whose depression was remitted at time of imaging and those who were unremitted, the shape comparison showed remitted subjects to be indistinguishable from controls (both sides) while the unremitted subjects differed in the midbody and the lateral side near the head. Hippocampal volume showed no difference between controls and remitted subjects but nonremitted subjects had significantly smaller left hippocampal volumes with no significant group differences in the right hippocampus. These findings may provide support to other reports of neurogenic effects of antidepressants and their relation to successful treatment for depressive symptoms.

## Introduction

The hippocampus plays a significant role in emotion processing with neural connections to key brain regions, including the thalamus, amygdala, basal ganglia, and prefrontal cortex [1], [2]. Structural imaging studies examining hippocampal volumes in depressed adults have been mixed; some report that volumes are smaller in depressed groups [3]–[7], while others have not found such a difference [8]–[10]. A more consistent finding is that smaller hippocampal volumes are seen in subjects with recurrent depression [4], [11] or earlier age of depression onset [4], [10], [11], suggesting that duration of depression, particularly untreated depression [4], may affect hippocampal volumes.

Similar results are seen in studies specifically examining depressed elderly populations. Some have found that depressed elders exhibit smaller hippocampal volumes and early age of onset is associated with smaller volumes [12], [13], but others have not found such relationships [14]–[16]. Smaller hippocampal volumes also appear to be associated with a lower probability of remission of depression with treatment [17]. Further, smaller left hippocampus volumes can be associated with increased risk of dementia [18].

Thus, while the hippocampus plays a role in the broader neural circuit which modulates mood and emotion, volumetric differences have not been consistently identified in depressed populations. Although there may be many factors that contribute to these different conclusions, one factor may be that only specific subregions of the hippocampus are affected in depression. If this is correct, there may not be a difference between depressed and nondepressed subjects in hippocampal volume, but hippocampi from these two groups could differ in shape so that local features could be larger in the hippocampus but with compensating negative differences in other regions. Analyses of hippocampal shape have been successfully used in studies of schizophrenia [19]–[21] and Alzheimer's disease [22]–[24], and also in a study examining younger adults with depression [8]. In this study we examined the shape-based differences between a group of subjects with late-life depression and a group of non-depressed comparison subjects.

## Methods

### Subjects and Clinical Evaluation

All subjects were recruited from individuals enrolled in the Conte Center for the Neuroscience of Depression at Duke University. Subjects were age 60 years or older; exclusion criteria included psychiatric diagnoses other than Major Depressive Disorder, including substance abuse or dependence, primary neurological disease including dementia, and contraindications to magnetic resonance imaging (MRI). The presence of comorbid generalized anxiety disorder symptoms did not prohibit enrollment if the evaluating clinician determined they were secondary to the depression diagnosis.

At time of enrollment into the Conte Center, depressed subjects met DSM-IV criteria for Major Depression. This diagnosis, and the absence of exclusionary diagnoses, was evaluated with the NIMH Diagnostic Interview Schedule (DIS) [25] which assessed major depression and age of onset of first depressive episode, enriched with items assessing lifetime history of psychosis, mania, anxiety disorders, and substance abuse or dependence. The presence of a diagnosis of Major Depression and absence of other psychiatric diagnoses including post-traumatic stress disorder were confirmed through a clinical interview with a geriatric psychiatrist. The clinical interview also assessed for a history of dementia or cognitive and functional deficits supporting such a diagnosis. Individuals with a diagnosis of dementia or where it was suspected were not enrolled.

Nondepressed control subjects were community volunteers with a non-focal neurological examination and no evidence for depression or other neuropsychiatric disease on the DIS. The study was approved by the Duke University Health System Institutional Review Board, and all subjects provided written informed consent.

After enrollment in the Conte Center, depressed subjects received antidepressant treatment provided by a study geriatric psychiatrist. This algorithm-based treatment was personalized to the individual subject and followed the Duke STAGED approach [26]. In this treatment algorithm, all commercially available antidepressant medications were available. Typically treatment begins with a selective serotonin reuptake inhibitor, unless an individual's past history shows a history of lack of response or intolerance to that class of drug. Antidepressant medication use in the month prior to MRI was reviewed.

In addition to the use of the DIS for confirming the clinical diagnosis of Major Depression, demographic data was obtained through subject interview. Depression severity at time of MRI was assessed using the clinician-rated Montgomery-Asberg Depression Rating Scale (MADRS) [27]. The Mini-Mental State Examination (MMSE) [28] was used to assess global cognitive function; subjects who scored less than a 24 were excluded.

### Image Acquisition

All subjects were screened for any condition where MRI was contraindicated. Subjects were imaged with a 3.0 Tesla whole-body MRI system (Trio, Siemens Medical Systems) using an 8-channel, receive-only volumetric radiofrequency coil. Padding was used to minimize motion of the head. The scanner alignment light was used to adjust the head tilt and rotation so that the axial plane lights were aligned just above the orbits and the sagittal lights were aligned with the center of the nose. A rapid three-plane localizer scan was acquired to confirm the alignment. The imaging protocol included a 3-dimensional, T1-weighted turbo-flash pulse sequence with imaging parameters: TR/TE/Flip = 22/7/25°, 256 mm field-of-view, 160 slices, 1 mm by 1 mm by 1 mm cubic voxel dimension, pixel bandwidth = 100 Hz. This data was used to estimate the hippocampus boundary as discussed below.

### Hippocampus Boundary Determination

The MR images were transferred to the Neuropsychiatric Imaging Research Laboratory (NIRL), located at Duke University Medical Center, for processing. Hippocampal volumes were determined using the GRID Program that was developed at NIRL and has been described previously [29], [30]. GRID allows for semi-automated region tracing and determination of region-of-interest (ROI) volumes and boundaries.

The method for defining the hippocampal perimeter has been previously described [29]. On all slices, tracing began along the most inferior border of the main body of the hippocampus, and then moved laterally along the border between the hippocampus and the inferior lateral ventricles. Along the medial and superior borders, tracing included any thin strips of white matter along the lateral or superior surface. Pockets of cerebrospinal fluid were excluded; blood vessels were transected unless they were prominent or did not extend into the hippocampal body. If motion, poor contrast, or other factors rendered any one slice unreadable, a volume for that slice was generated by averaging the volumes from the previous and subsequent slices. If the first or last slices were unreadable, or if two middle slices were unreadable, the subject was excluded from analysis.

On each scan, tracing began with the most posterior coronal slice, then proceeded anteriorly. Measurement of the hippocampus began when the pulvinar nucleus of the thalamus obscured the crura fornicis; if the crus was only obscured on one side, then only that side was measured. On the first few slices, the lateral body of the hippocampus appears as a rough oval, which narrows medially into a thin strip of gray matter that curves downwards along the border of the cistern. The fimbria, which extends from the superior surface of the hippocampus across the CSF into the white matter above, was transected at its narrowest point. Along the medial border of the hippocampus, the thin strip of gray matter was cut at its narrowest point, and tracing then continued around the hippocampal body to the starting point.

On more anterior slices, the amygdala begins to appear just superior to the hippocampus, which roughly resembles a kidney in shape, with no external connections. The amygdala-hippocampal transition zone appears as a diffuse area of gray matter between the anterior portion of the hippocampus and the posterior portion of the amygdala; as with the fimbria, this area was transected at its narrowest point, which was usually found between the inferior lateral ventricles and the cistern. Continuing anteriorly, the inferior lateral ventricles gradually shift from a vertical to a horizontal orientation, but remain superior to the hippocampus. The anterior border of the hippocampus was defined as the slice on which the inferior lateral ventricles appeared horizontally without any body of gray matter visible below them.

All image analysis technicians received extensive training by experienced analysts. Reliability was established by repeated measurements on 10 MR scans. Intraclass correlation coefficients (ICC's) attained were as follows: left hippocampus = 0.899, right hippocampus = 0.798.

### Hippocampus Shape Analysis

**Figure 1:**
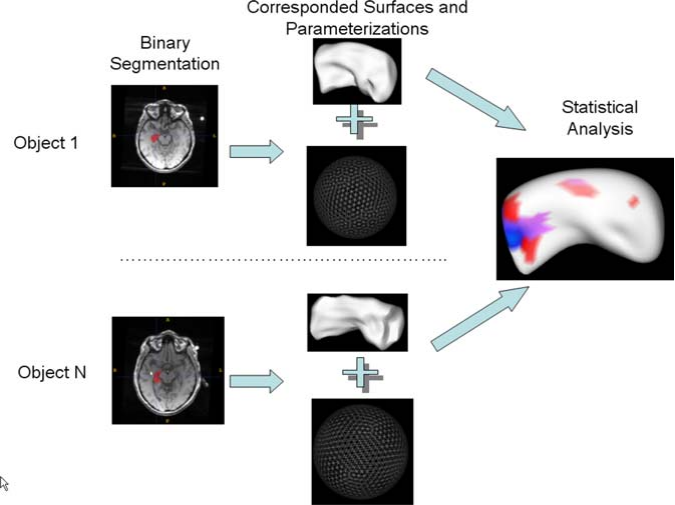
Shape analysis process.

The 3D hippocampal shape analysis is based on the use of spherical harmonic basis functions (SPHARM) to fit the hippocampus boundary. The shape analysis algorithm used in this study was proposed and described in detail in [20], [31]. A schematic view of the shape analysis process is shown in Figure 1.

The first step in the algorithm is to identify the correspondences among the surfaces of hippocampi. At first, the binary images that contain the surface points that were determined by the boundary identification process described above were pre-processed to fill small holes and minimally smoothed. At this point, then, the surface was described by the vertexes of the (cubic) voxels on the surface. A triangulation mesh was then generated from each binary image by dividing each exterior voxel face into two triangles. A spherical parameterization was then computed for the triangulation mesh using a distortion minimizing spherical mapping. Finally, the SPHARM coefficients were computed from the mesh and its parameterization using straightforward fitting to spherical harmonic functions up to the twelfth degree. The cut-off at the twelfth degree was determined by examination of residuals to the fits to have the lowest degree that would adequately describe the shape. By aligning the first order ellipsoids of all the parameterizations, the correspondences across all surfaces were established. All parameterizations were sampled into triangulated meshes. The triangulated meshes were then spatially aligned using a rigid transformation. To offset the effects of different head sizes, the surfaces were scaled according the corresponding head size.

### Statistical Methods

All statistical analyses, except shape analyses, were performed using SAS 8.2 (SAS Institute, Cary, NC). Demographic variables and clinical measures were compared using pooled, two-tailed *t*-tests for continuous variables and chi-square models for categorical variables. Analysis of group differences in left and right hippocampal volume were modeled using the GLM procedure, which covaried for age, sex, and total cerebral volume.

Using a MADRS score of less than 10 as a definition of remission [32], we then dichotomized the depressed cohort into those currently remitted, and those who were not. This resulted in a trichotomous diagnostic variable (nondepressed, depressed-remitted, and depressed-nonremitted). We tested for differences between comparison subjects, remitted depressed, and nonremitted depressed subjects using analysis of covariance (ANCOVA). We repeated the previous models, replacing the previous dichotomous diagnosis variable with this new trichotomous variable. Finally, we used the least squared means approach to identify significant differences between groups.

For the shape analysis statistical methods, the final shape description consisted of the coordinates of the corresponding points (appropriately scaled). The null hypothesis is that the distribution of the spatial locations at each surface point is the same for every subject regardless of the group. Group differences between two groups of surfaces were tested using a multivariate metric: Hotelling's T-squared test which is a multivariate version of Student's t statistic. The analysis generated a raw (unadjusted for multiple comparisons) significance map showing raw p-values exceeding 0.05 across the surface. The distance of the two mean surfaces of the two groups were also generated.

To quantitatively measure the global shape difference, the global average deviation between the mean surfaces of the two groups as well as the near maximal distance (95%) between the mean surfaces of the two groups (average and maximal distance across the whole surface) was calculated and tested with a two tailed t-test. This analytic approach was used first for comparisons between depressed and nondepressed comparison subjects, but then also paired comparisons between nondepressed, depressed remitted and depressed non-remitted subjects.

**Figure 2:**
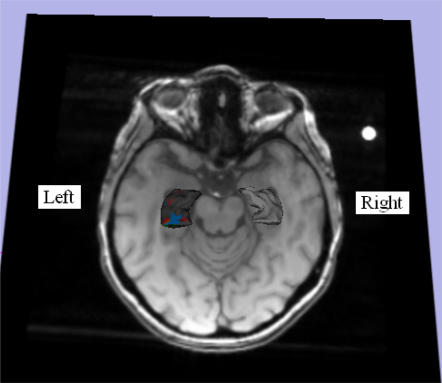
The analysis of hippocampus: control vs. depressed. The distance maps masked by the significance maps are shown on a pair of representative hippocampi. The scale of color code is illustrated in a color bar and in millimeters.

**Figure 3:**
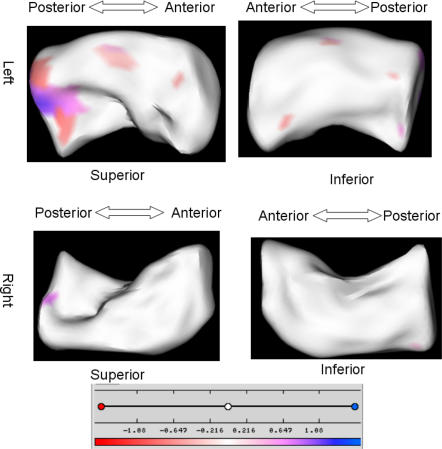
A pair of representative hippocampi are along with the T1 MRI scan from which the hippocampi were segmented.

To present the shape differences we incorporated our results into a single informative visualization shown in Figure 2 and Figure 3, where the maps in Figure 3 that show the distance from the mean surface of the group A to the mean surface of the group B are masked by the significance maps, and displayed in anatomical context in a corresponding T1-weighted MRI scan in Figure 2. In this study, group A was the control, group B was the depressed patients. Thus, this visualization shows three kinds of information at a specific location on the surface:

1. *The magnitude of the distance between the mean shapes of group A and group B:* White color is used where the magnitudes are zero. The color gets darker as the magnitudes of the distance increase. The magnitude is in millimeters.
2. *The direction of the distances:* The distance is positive if the mean surface of group A is protruding outside of the mean surface of group B; the distance is negative if the mean surface of group A is shrinking below the mean surface of group B. Positive distances are color-coded by blue and negative distances are color-coded by red.
3. *The significance map:* The distances are set to zero wherever the raw p-value exceeds 0.05 and the color is set to white at that location. In other words, surface locations are in white if the differences are not statistically significant regardless the magnitude of the distance. Therefore, only the distances which are statistically significant are visualized in this illustration.

## Results

This sample included 43 nondepressed comparison subjects and 61 depressed subjects. There was no difference in sex representation (depressed: 60.7% female, 37/61; nondepressed: 67.4% female, 29/43; χ^2^ = 0.5009, *p* = 0.4791), although the nondepressed population was older (depressed: 65.9y, SD = 5.5y; nondepressed: 69.0y, SD = 5.5y; t = 2.87, 102 *df*, *p* = 0.0048). There was no difference in MMSE score between the groups (depressed: 28.4, SD = 2.2; nondepressed: 28.7, SD = 1.5; t = 0.89, 102 *df*, *p* = 0.3748). The depressed cohort on average had a mild level of depression severity at time of imaging (MADRS = 16.1, SD = 10.7, range 0–44), with a mean age of first depressive episode onset of 39.3 years (range = 4–76 years, SD = 19.9 years).

We also reviewed what antidepressant medications depressed subjects had been taking over the month before the MRI. 29 (47.5%) were taking a serotonin reuptake inhibitor (SSRI), 15 (24.6%) were taking venlafaxine, 9 (14.8%) were taking bupropion, 5 (8.2%) were on nortriptyline, 1 (1.6%) was on mirtazapine, and 7 (11.5%) were not on an antidepressant medication. 5 people (8.2%) were on two antidepressants. Per exclusion criteria, no nondepressed comparison subject was taking an antidepressant.

Depressed subjects generally exhibited smaller hippocampal volumes than did nondepressed subjects, although in univariate analyses of unadjusted volumes, these differences were not statistically significant (left hippocampus: depressed = 3.42mL, SD = 0.54mL; nondepressed = 3.55mL, SD = 0.48mL; t = 1.29, 102 df, p = 0.1994; right hippocampus: depressed = 3.65mL, SD = 0.55mL; nondepressed = 3.66mL, SD = 0.57mL; t = 0.08, 102 df, p = 0.9236). The models controlling for age, sex, and total cerebral volume showed a significant difference between depressed and nondepressed subjects in left (F_1,103_ = 5.26; *p* = 0.0240) but not right hippocampus volume (F_1,103_ = 0.41; *p* = 0.5213).

The visualization demonstrating group differences between control and depressed subjects is overlaid on a pair of representative hippocampi in Figure 3. The regional differences are most noticeable in the left hippocampus, on the superior side, approximately midway down the body. The area where there is a shape difference shows a central region of contraction (smaller in depressed than nondepressed subjects) flanked by regions of expansion (larger in depressed than nondepressed subjects). Although the location of these anatomic changes cannot be specifically pinpointed, in the depressed cohort these changes correspond to a contraction in the dentate gyrus and possibly CA4 region, with areas of expansion in the subiculum and the CA2-3 region.

The global shape difference metrics yielded no significant difference in the average difference or the maximal deviation between the average surface for the two groups (p>0.1 for all measures for left and right hippocampus) indicating that the differences shown are local.

### Three-Way Comparisons

To further investigate the shape difference of remitted patients, depressed subjects were divided into two groups: currently remitted (N = 24) and non-remitted (37). See Table 1 for demographic differences between these two groups and nondepressed comparison subjects. Of the remitted subjects, 13 (54.2%) were on a SSRI, 5 (20.8%) on venlafaxine, 2 (8.3%) on bupropion, 2 (8.3%) on nortriptyline, and 1 (4.2%) was on no antidepressant, with 1 person on two agents. Of the nonremitted subjects, 16 were on a SSRI (43.2%), 10 (27.0%) on venlafaxine, 7 (18.9%) on bupropion, 3 (8.1%) on nortriptyline, 1 (2.7%) on mirtazapine, and 4 (10.8%) on no antidepressant, with 4 people on two agents. Using Fisher's exact test, there was no difference in medication use between the remitted and nonremitted groups (*p* = 0.8541)

**Table 1:** Group differences based on diagnosis and remission status

	Nondepressed (N = 43)	Depressed–Remitted (N = 24)	Depressed–Nonremitted (N = 37)	*df*	Test statistic	*p* value
Age (y)	69.0 (5.5)	66.9 (5.7)	65.1 (5.3)	2, 103	F = 4.91	0.0092
Sex (% female)	67.4% (29/43)	62.5% (15/24)	59.5% (22/37)	2	χ^2^ = 0.51	0.7754
MMSE	28.7 (1.5)	28.3 (3.0)	28.4 (1.8)	2, 103	F = 0.40	0.6686
MADRS	-	2.0 (2.5)	22.3 (6.4)	49.2	t = 17.10	<0.0001
Age of Onset (y)	-	41.5 (21.7)	38.1 (19.1)	59	t = 0.56	0.5783
Hippocampus Volume (mL)						
- Left	3.55 (0.48)	3.51 (0.62)	3.36 (0.48)	2,103	F = 1.42	0.2474
- Right	3.65 (0.55)	3.72 (0.59)	3.60 (0.53)	2, 103	F = 1.39	0.2541
Cerebrum Volume (mL)	1571.2 (160.9)	1527.5 (182.7)	1562.9 (171.7)	2, 103	F = 0.55	0.5800

MMSE = mini-mental state exam; MADRS = Montgomery-Asberg Depression Rating Scale. All measures presented as mean (standard deviation), except sex. All volumes presented in milliliters. Differences between groups tested using analysis of covariance (ANCOVA), except for sex, which used the chi square test. Age of onset data were examined using two-tailed pooled t-tests and MADRS data using a Satterthwaite t-test due to unequal variances. Age of onset and MADRS data were only available for the depressed population.

When the trichotomous diagnostic variable was incorporated into models controlling for age, sex, and total cerebral volume, we saw no significant relationship between this variable and right hippocampal volume (F_1,103_ = 2.27, *p* = 0.1083). However, there was a statistically significant effect of group in the left hippocampus (F_1,103_ = 5.77, *p* = 0.0043). Using the least squares means analysis, nondepressed subjects exhibited the largest adjusted mean left hippocampus volume (3.59mL), while remitted depressed subjects were slightly smaller (3.54mL) and nonremitted depressed subjects exhibited the smallest mean volume (3.29mL). Pairwise comparisons demonstrated that this difference was statistically significant between nondepressed and depressed-nonremitted subjects (*p* = 0.0017), and between depressed-remitted and depressed –nonremitted subjects (*p* = 0.0159), but not between nondepressed and depressed-remitted cohorts (*p* = 0.6358).

**Figure 4:**
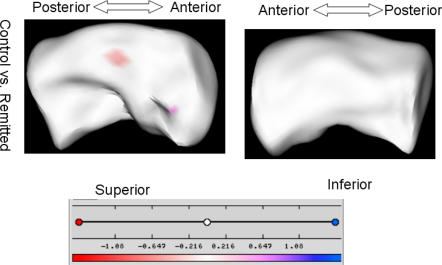
The analysis of left hippocampus: control vs. remitted. The distance maps masked by the significance maps are shown on a representative hippocampus. The scale of color code is illustrated in a color bar and in millimeters.

**Figure 5:**
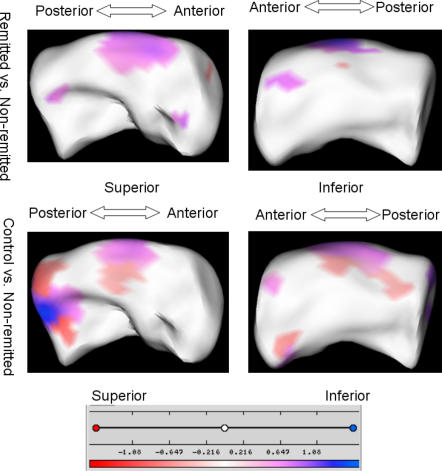
The analysis of left hippocampus: remitted vs. non-remitted, control vs. non-remitted. The distance maps masked by the significance maps are shown on a representative hippocampus. The scale of color code is illustrated in a color bar and in millimeters.

The visualizations demonstrating group differences are shown in Figure 4 and Figure 5. There were no statistically significant differences in shape in the right hippocampus, so only left hippocampal differences are shown. These analyses primarily demonstrate a difference in two regions. The first was the same region identified in the comparison between depressed and nondepressed control subjects, occurring on the superior surface midway down the body. Here we see that the same statistically significant pattern of a central contraction flanked by two regions of expansion observed between depressed and control subjects is observed between nonremitted-depressed and control subjects, but not between control and remitted subjects, or remitted and nonremitted subjects.

The second large region exhibiting statistically significant differences in shape is closer to the head, along the lateral boundary. Here we see that when compared with both remitted subjects and control subjects, non-remitted subjects exhibit contraction of this region, which would roughly correspond to regions CA1 and CA2. This difference was not observed between remitted and control subjects.

## Discussion

Although this is not the first study to utilize shape analyses of the hippocampus in either depression [8] or in an elderly population [22], [33], to our knowledge it is the first to use this technique in an older population of depressed and nondepressed subjects. In this study, depressed subjects exhibited both a volumetric difference and a shape difference in the left hippocampus. This was primarily due to differences in the nonremitted depressed group, while there was no difference in hippocampal shape or volume between the control group and remitted depressed group. Given how we approached this study with no a priori hypotheses about where we would expect to see changes, this was an exploratory study and these results should be considered as being hypothesis-generating.

Numerous studies have examined hippocampal volume in geriatric depression. Some have reported that depressed elders exhibit smaller hippocampal volumes [34]–[36], primarily in the right hippocampus [12], [13], [37], while others have found no difference between depressed and nondepressed elderly cohorts [16], [38]. In contrast, our current report identified a reduction in the volume of the left hippocampus, but not the right. Possible explanations for these conflicting findings include methodological issues, such as different hippocampal boundary definitions, or differences in image acquisition–such as our use of 3T MRI, which offers improved image resolution. Differences in clinical factors also become important, such as differences in age, duration of depression [4], or antidepressant treatment [39].

Far fewer studies have examined hippocampal shape. A study of hippocampal shape in a younger adult cohort of depressed and nondepressed subjects identified depression-related deformation of the subiculum [8]. Our study excluded subjects with dementia, but finding of differences in the subiculum have also been observed in subjects with dementia of Alzheimer's type (DAT) [24], although DAT subjects exhibit more widespread changes in the CA1 region, which encompasses much of the head and lateral aspect of the hippocampus. DAT may also be associated with changes in the dentate gyrus, and possibly parts of CA2 or CA3 [22], which is comparable to our current findings. Also similar to our findings, shape differences in DAT may be more apparent in the left hippocampus than right [22], [23].

These similarities support theories associating depression and DAT. There are a number of studies supporting an association between a lifetime history of depression with an increased risk of DAT [40]–[44]. Both duration and number of depressive episodes is associated with smaller hippocampal volumes [4], [12], while smaller left hippocampal volume in older depressed individuals is associated with a greater risk of dementia [18]. Moreover, a lifetime history of depression is associated with greater DAT-related neuropathological changes in the hippocampus [45]. Given how the risk of DAT increases with age, this also raises the issue that there was a significant difference in age between our diagnostic groups. Since the mean difference was only 3.1 years, and the nondepressed cohort was older, it is unlikely that the age difference is responsible for the observed shape differences. However, there is the possibility the study included subjects who had no obvious clinical symptoms of Alzheimer disease but were early in the process of its development.

The majority of volumetric and shape differences observed between depressed and nondepressed subjects appear to be primarily driven by the nonremitted depressed group. In analyses where the depressed cohort was divided into remitted and nonremitted subjects, there were widespread differences between nonremitted and control subjects, and nonremitted and remitted subjects, but only isolated, small differences in shape between remitted and control subjects. Although there are no previously published analyses of hippocampus shape between remitted and nonremitted depressed subjects, our volumetric findings are similar to previously reported volumetric studies [5], [46].

As this is a cross-sectional study, we cannot determine the causal relationship between remission status and our hippocampus finding. Individuals with greater hippocampal abnormalities may be less likely to achieve remission [17]. Alternatively, remission of depression may be associated with correction of the hippocampal shape and volumetric differences seen in depressed subjects who are currently symptomatic. If this second hypothesis is correct, this correction of hippocampal structure may be secondary to antidepressants, which are thought to affect hippocampal volume through neurogenesis [39], although this has not been conclusively demonstrated [47]. It is possible that antidepressant use in particular may be related to our findings of a depression-related expansion seen in the vicinity of the dentate gyrus. The dentate gyrus is a specific region of the hippocampus that continues to give rise to new neurons throughout adult life; this production may be inhibited by stress or increased glucocorticoid levels, while trophic factors and serotonin may increase this production [39]. Thus our findings of contraction of the dentate gyrus in nonremitted depressed subjects when compared with control subjects, while remitted subjects do not differ from control subjects, may be related to the neurotrophic effect of antidepressant medications when accompanied by remission of symptoms. Clearly such a hypothesis requires further study.

The study has limitations that should be noted, including our definition of the hippocampus. Our measure does not include the more posterior component of the body nor the tail. Thus our findings are only applicable to the head and anterior body. A second limitation of our results is the absence of a correction for multiple comparisons. With our image analysis technique, methods correcting for multiple comparison based family wise errors often result in a major overcorrection and highly reduced sensitivity, as they eliminate many effects that are found at the *p*<0.05 level. However the hippocampal volumetric measures reported do not have this limitation and support the shape conclusions. Even with this potential limitation, the shape results we are reporting should be of strong interest to the community to illustrate the potential of shape analysis methods and despite being exploratory, these results provide crucial data for future studies. A third limitation was in the clinical assessments, as there was not a more thorough analysis of depression history, such as lifetime duration of depressive symptoms, which itself has been associated with hippocampal volume differences [4]. Although we saw no significant difference between remitted and nonremitted subjects on age of depression onset or current antidepressant use, these assessments do not capture the longer term duration of depression. Additionally, the samples were not matched for potential differences such as handedness, and even differed in age which itself has an effect on brain structure.

This study demonstrates a left-hemisphere difference in hippocampal shape between elderly depressed and nondepressed subjects, primarily due to the effect of the nonremitted depressed subjects in our cohort. These differences may be related to the relationship between depression and risk of dementia or historical course of depression. Moreover, genetic polymorphisms of the serotonin transporter-linked promoter region (5HTTLPR) and brain derived neurotrophic region (BDNF) genes have been linked with depression and hippocampal volume differences, and should be considered in future studies as these and similar polymorphisms may have contributed to the differences observed in this study.. Further work using longitudinal designs, with comprehensive assessments of depression history, and in subjects who are antidepressant-free, is needed to better examine the hypotheses generated by this study.
